# Sulforaphane Increase Mitochondrial Biogenesis-Related Gene Expression in the Hippocampus and Suppresses Age-Related Cognitive Decline in Mice

**DOI:** 10.3390/ijms23158433

**Published:** 2022-07-29

**Authors:** Sunao Shimizu, Shuya Kasai, Hiromi Yamazaki, Yota Tatara, Junsei Mimura, Máté János Engler, Kunikazu Tanji, Yoshikazu Nikaido, Takuro Inoue, Hiroyuki Suganuma, Koichi Wakabayashi, Ken Itoh

**Affiliations:** 1 Innovation Division, KAGOME Co., Ltd., 17 Nishitomiyama, Nasushiobara 329-2762, Tochigi, Japan; sunao_shimizu@kagome.co.jp (S.S.); takuro_inoue@kagome.co.jp (T.I.); hiroyuki_suganuma@kagome.co.jp (H.S.); 2 Department of Vegetable Life Science, Hirosaki University Graduate School of Medicine, 5 Zaifu-cho, Hirosaki 036-8562, Aomori, Japan; kasai-s@hirosaki-u.ac.jp (S.K.); yamazaki.hiromi@fbri.org (H.Y.); ytatara@hirosaki-u.ac.jp (Y.T.); jmimura@hirosaki-u.ac.jp (J.M.); 3 Department of Stress Response Science, Center for Advanced Medical Science, Hirosaki University Graduate School of Medicine, 5 Zaifu-cho, Hirosaki 036-8562, Aomori, Japan; mamorute24@gmail.com; 4 Department of Neuropathology, Institute of Brain Science, Hirosaki University Graduate School of Medicine, 5 Zaifu-cho, Hirosaki 036-8562, Aomori, Japan; kunikazu@hirosaki-u.ac.jp (K.T.); koichi@hirosaki-u.ac.jp (K.W.); 5 Department of Metabolomics Innovation, Hirosaki University Graduate School of Medicine, 5 Zaifu-cho, Hirosaki 036-8562, Aomori, Japan; ynikaido@hirosaki-u.ac.jp; 6 Department of Anesthesiology, Hirosaki University Graduate School of Medicine, 5 Zaifu-cho, Hirosaki 036-8562, Aomori, Japan

**Keywords:** NRF2, sulforaphane, glucoraphanin, cognitive decline, PGC1α, TFAM, mitochondrial biogenesis

## Abstract

Sulforaphane (SFN) is a potent activator of the transcriptional factor, Nuclear Factor Erythroid 2 (NF-E2)-Related factor 2 (NRF2). SFN and its precursor, glucoraphanin (sulforaphane glucosinolate, SGS), have been shown to ameliorate cognitive function in clinical trials and *in vivo* studies. However, the effects of SGS on age-related cognitive decline in Senescence-Accelerated Mouse Prone 8 (SAMP8) is unknown. In this study, we determined the preventive potential of SGS on age-related cognitive decline. One-month old SAMP8 mice or control SAM resistance 1 (SAMR1) mice were fed an ad libitum diet with or without SGS-containing broccoli sprout powder (0.3% *w*/*w* SGS in diet) until 13 months of age. SGS significantly improved long-term memory in SAMP8 at 12 months of age. Interestingly, SGS increased hippocampal mRNA and protein levels of peroxisome proliferator-activated receptor gamma coactivator-1 alpha (PGC1α) and mitochondrial transcription factor A (TFAM), which are master regulators of mitochondrial biogenesis, both in SAMR1 and SAMP8 at 13 months of age. Furthermore, mRNAs for nuclear respiratory factor-1 (NRF-1) and mitochondrial DNA-encoded respiratory complex enzymes, but not mitochondrial DNA itself, were increased by SGS in SAMP8 mice. These results suggest that SGS prevents age-related cognitive decline by maintaining mitochondrial function in senescence-accelerated mice.

## 1. Introduction

Neurodegenerative dementia such as Alzheimer’s disease is a progressive neurodegenerative disease for which no fundamental treatment has been found. Oxidative stress may play a role in the onset of dementia, whereas a reduction in oxidative stress may be effective for preventing and treating dementia [1]. Transcription factor Nuclear Factor E2-Related factor 2 (NRF2, gene name *Nfe2l2*) regulates the expression of phase 2 detoxifying enzymes and antioxidant proteins, such as heme oxygenase-1 (HO-1), through binding to the antioxidant responsive element (ARE) [2]. It also regulates genes associated with proteasomes, autophagy, anti-inflammation, nerve growth factor signaling, and mitochondrial quality control. Thus, NRF2 is a putative target for the prevention of dementia and in fact, NRF2 activation has been shown to enhance cognitive function in several animal models [3]. For example, activation of NRF2 reduced neuroinflammation and improved cognitive function in the NL-G-F mouse model of Alzheimer’s disease (AD) [4]. In addition, eight weeks of lycopene administration in ICR mice attenuated the decline in cognitive function induced by D-galactose administration through the NRF2 pathway [5]. These results suggest that the oral administration of NRF2 activators can maintain and improve cognitive function and prevent AD; however, the details of this mechanism need to be clarified. 

Reduced mitochondrial function may be one cause of AD. For example, the activity of mitochondrial respiratory chain complex IV (cytochrome c oxidase) was shown to be significantly decreased in the hippocampus of AD patients [6]. In addition, the activity of cytochrome c oxidase was decreased and reactive oxygen species (ROS) were increased in a human teratoma cell line (NT2) with mitochondrial DNA (mtDNA) from an AD patient compared with mtDNA transfected from an age-matched healthy person [7]. Mitochondria produce ROS during electron transfer through the mitochondrial respiration chain [8]. When mitochondrial function is impaired, increased ROS is released, which can cause damage to surrounding tissues. Mitophagy constantly removes dysfunctional mitochondria, which are replaced with new mitochondria by mitochondrial biogenesis [9]. Importantly, mitochondrial biogenesis is regulated by the peroxisome proliferator-activated receptor gamma coactivator-1 alpha (PGC1α), nuclear respiratory factor-1 (NRF-1, gene name *Nrf1*), and mitochondrial transcription factor A (TFAM) pathways. PGC1α stimulates mitochondrial biogenesis and respiration through *Nrf1* gene expression. NRF-1 binds to the *Tfam* promoter to drive transcription and replication of mitochondrial DNA [10]. NRF2 may regulate mitochondrial biogenesis by interacting with PGC1α [11]. For example, activation of mitochondrial biogenesis was suppressed in NRF2-deficient mice in a mouse model of acute lung injury [12]. Therefore, NRF2 activation may inhibit the loss of mitochondrial function, which is widely observed in neurodegenerative diseases.

Some cruciferous plants are rich in NRF2 activators, of which a representative example is sulforaphane (SFN), a metabolite of glucoraphanin (sulforaphane glucosinolate; SGS) found in broccoli sprouts (BS) [13]. Administration of SFN improved cognitive function in PS1V97L transgenic (Tg) AD model mice [14,15] and ameliorated memory impairment in streptozotocin-mediated diabetic rats [16] and okadaic acid-treated rats [17]. Furthermore, the addition of sulforaphane activated mitochondrial biogenesis [18]. Small scale human clinical trials using SGS resulted in the improvement of symptoms of patients with schizophrenia and autism spectrum disorders [19]. Furthermore, a 12-week supplementation of SGS ameliorated age-related cognitive decline in healthy elderly adults [20]. Senescence-Accelerated Mouse Prone 8 (SAMP8), generated by crossing inbred mouse strains, results in an accelerated aging phenotype and has been used as a model in many studies of cognitive function. SAMP8 exhibits reduced nuclear NRF2 protein expression in the hippocampus compared with its control strain, SAM resistance 1 (SAMR1). Further genetic modification of the brain to reduce NRF2 exacerbates the level of neural inflammation and accelerates age-related cognitive decline [21]. 

In the present study, we determined whether long-term dietary intake of SGS ameliorated age-related cognitive decline through NRF2 activation and improvement of mitochondrial function in SAMP8 mice compared with control SAMR1 mice.

## 2. Results

### 2.1. Co-Treatment with a Mixture of SGS and Mustard Extracts Efficiently Induces NRF2 Target Gene Expression in C57BL/6J Mice

**Figure 1:**
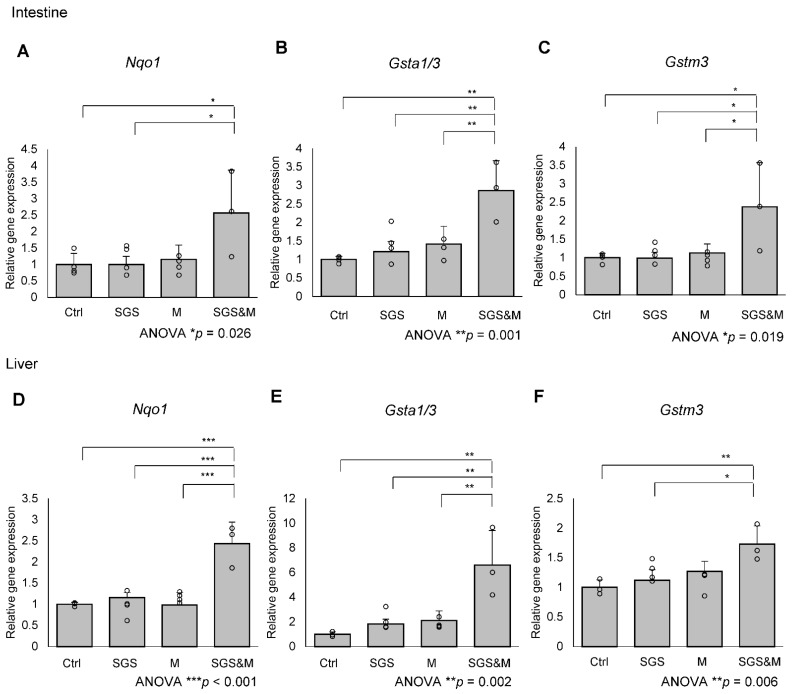
Oral administration of broccoli sprout and mustard activates NRF2 target genes in the intestine and liver. C57BL/6J mice were treated control diet (Ctrl) or diet supplemented with 0.3% (*w*/*w*) glucoraphanin (sulforaphane glucosinolate, SGS), 1% (*w*/*w*) mustard (M), or both 0.3% (*w*/*w*) SGS and 1% (*w*/*w*) mustard (SGS&M) for a week. Expression levels of NRF2 target genes, which were (**A**) *Nqo1*, (**B**) *Gsta1/3* or (**C**) *Gstm3*, in the intestine or (**D**–**F**) liver were quantified by qPCR. The value for control food treated mice was set to 1, and relative expressions are shown as the mean folds + SD from multiple independent animals (Ctrl: n = 4, SGS: n = 4, M: n = 4, SGS&M: n = 3 for intestine. Ctrl: *n* = 3, SGS: *n* = 4, M: *n* = 4, SGS&M: *n* = 3 for liver.). * *p* < 0.05, ** *p* < 0.01, *** *p* < 0.001 compared with the control group (one-way ANOVA with Tukey-Kramer post hoc test).

As we extracted SGS from broccoli sprout in boiling water, myrosinase in the sprout must be inactivated [22]. In this study, we added mustard as a supplier of myrosinase. First, we examined whether myrosinase in mustard actually converts SGS to SFN in wild-type C57BL/6J mice as a basis for the activation of the NRF2 pathway. Wild-type C57BL/6J were fed an ad libitum diet with or without 0.3% SGS or mustard for one week. Treatment of mice with SGS or mustard alone did not induce NRF2 target genes in the liver or the intestine, but co-treatment of mice with a mixture of SGS and mustard efficiently induced NRF2 target gene expression, indicating the efficient conversion of SGS to SFN by mustard myrosinase (Figure 1).

### 2.2. SGS Intake Prevents Age-Related Cognitive Decline in SAMP8

**Figure 2:**
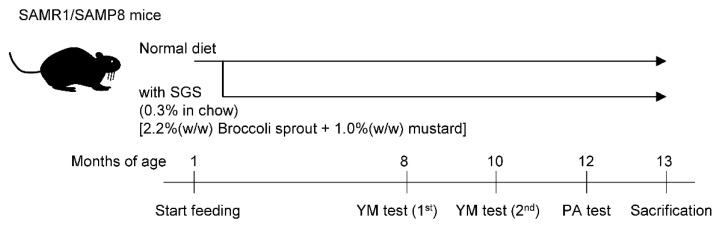
Experimental scheme. Both SAMR1 and SAMP8 mice were fed an ad libitum diet with or without 0.3% SGS for 12 months. Mice underwent a modified Y-maze (YM) test at 8 and 10 months of age and a step-through passive avoidance test (PA) at 12 months of age. The mice were then sacrificed and their hippocampus was removed at 13 months of age.

**Figure 3:**
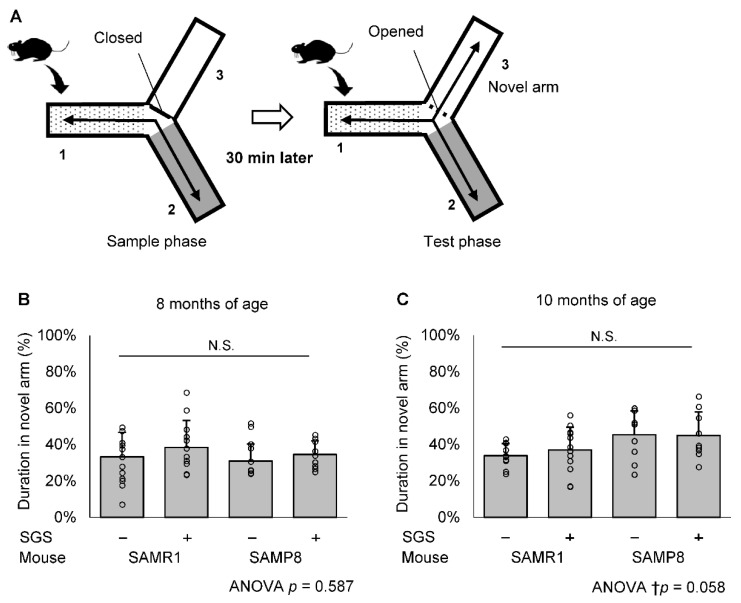
The modified YM test. (**A**) Schematic drawings of the YM test and the experimental procedures. (**B**,**C**) The duration in the novel arm during the test phase was represented by short-term memory. Individual values of the mise are shown as open circles with means + SD of SAMR1/SGS− (*n* = 12), SAMR1/SGS+ (*n* = 12), SAMP8/SGS− (*n* = 10) and SAMP8/SGS+ (*n* = 9) at 8 months of age (**B**) and SAMR1/SGS− (*n* = 10), SAMR1/SGS+ (*n* = 12), SAMP8/SGS− (*n* = 10), and SAMP8/SGS+ (*n* = 9) at 10 months of age (**C**). The data were analyzed by ANOVA. *N.S.*, not significant.

**Figure 4:**
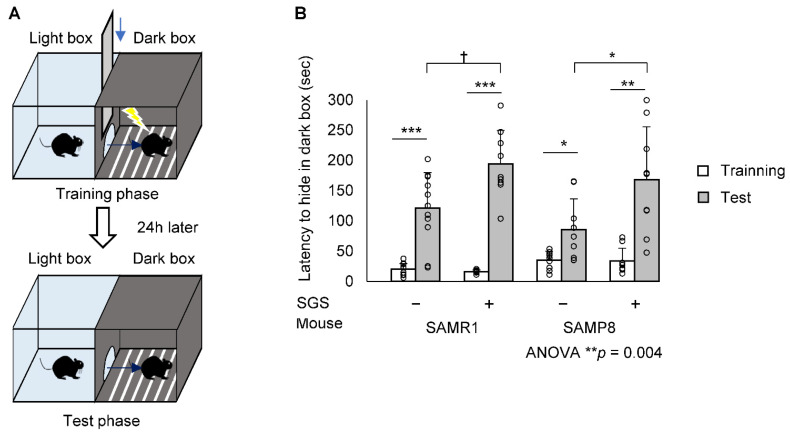
The step-through PA test. Both SAMP8 and SAMR1 mice underwent the step-through PA test at 12 months of age. (**A**) Schematic drawings of the PA test and the experimental procedures. (**B**) Step-through latency to the dark box represented long-term memory. Individual values of the mise are shown as open circles with means + SD of SAMR1/SGS− (*n* = 11), SAMR1/SGS+ (*n* = 9), SAMP8/SGS− (*n* = 9), and SAMP8/SGS+ (*n* = 9). The data were analyzed by ANOVA with Tukey-Kramer’s post hoc test for multiple comparisons or by a paired *t*-test for before-after comparisons of the same group. † *p* < 0.1, * *p* < 0.05, ** *p* < 0.01, *** *p* < 0.001.

The scheme for SGS treatment and the memory test is shown in Figure 2. We observed that SGS significantly reduced the body weight in SAMR1 during the time course. However, SGS significantly reduced the body weight in SAMP8 only during the early time period of th course (i.e., 6 to 10 months), but did not change the body weight after that period (Figure S1). To evaluate the effect of SGS plus mustard (hereafter simply called SGS) intake on short-term memory in SAMR1 and SAMP8 mice, we conducted a modified YM test at 8 and 10 months of age (Figure 3A). There was no significant difference in duration in the novel arm among the four groups at both 8 and 10 months of age (Figure 3B,C). Next, to evaluate the effect of SGS intake on long-term memory, we conducted a PA test at 12 months of age (Figure 4A). The latency to hide in the dark box was significantly increased after training in all groups. The latency in the test phase of the SGS-fed group was significantly longer in SAMP8 mice compared with the NC-fed group (*p* < 0.05. Figure 4B), but it only tended to be longer in the SAMR1 group compared with the NC-fed group (*p* = 0.081).

### 2.3. SGS Intake Differentially Modulates the Expression of NRF2/ARE Pathway Genes in the Hippocampi of SAMR1 and SAMP8 Mice

Hippocampal atrophy is associated with decreased memory function during normal aging [23,24,25]. We determined whether the preventive effect of SGS on long-term memory was derived either from structural or functional changes in the hippocampus. Neither hematoxylin and eosin staining nor Kluver-Barrera staining revealed structural change in the SAMR1 and SAMP8 hippocampus (Figure S2). Therefore, we hypothesized that SGS intake improved long-term memory by improving functional aspects of the hippocampal cells.

**Figure 5:**
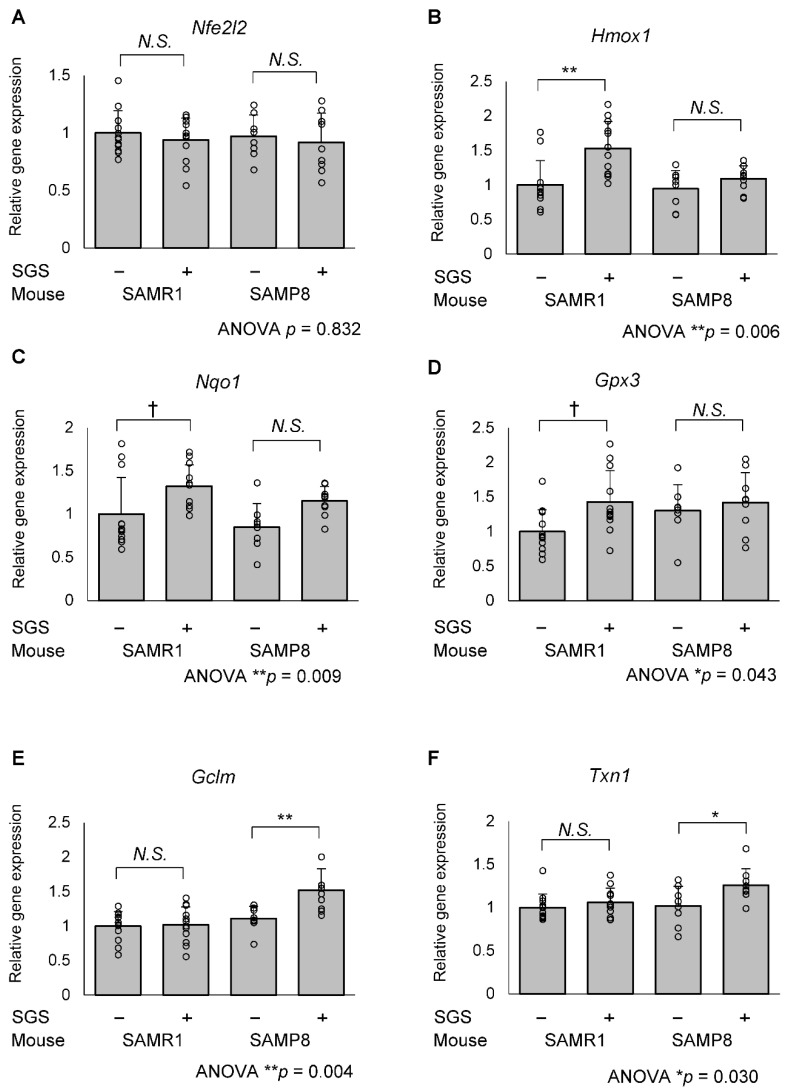
mRNA expression of NRF2/ARE-regulated genes in the SAMR1 or SAMP8 hippocampi. The SAMR1 and SAMP8 mice were fed a diet with or without SGS (0.3% *w*/*w*) from 1 to 13 months of age. The mice were dissected at 13 months of age and hippocampal mRNA was subjected to RT-qPCR analysis. The mRNA expression of the control-treated SAMR1 mouse was set as 1 and the relative mRNA expression of (**A**) *Nfe2l2*, (**B**) *Heme oxygenase 1* (*Hmox1*) (**C**) *NAD(P)H quinone dehydrogenase 1* (*Nqo1*) (**D**) *Glutathione peroxidase 3* (*Gpx3*) (**E**) *Glutamate-cysteine ligase modifier subunit* (*Gclm*) and (**F**) *Thioredoxin 1* (*Txn1*) were normalized to *Glyceraldehyde-3-phosphate dehydrogenase* (*Gapdh*). Individual values of the mise are shown as open circles with means + SD of SAMR1/SGS− (*n* = 12), SAMR1/SGS+ (*n* = 12), SAMP8/SGS− (*n* = 8), and SAMP8/SGS+ (*n* = 9). The data were analyzed by ANOVA with Tukey-Kramer’s post hoc test for comparing the differences. † *p* < 0.1, * *p* < 0.05, ** *p* < 0.01, *N.S.*, not significant.

To evaluate whether the oral administration of SGS activated NRF2 in the hippocampus, we measured the expression of *Nfe2l2* mRNA as well as NRF2-regulated genes. Although *Nfe2l2* mRNA levels were not significantly altered among the 4 groups, different subsets of NRF2-regulated genes were significantly elevated in SAMR1 and SAMP8 mice. In SAMR1 mice, the expression of *heme oxygenase 1* (*Hmox1*) in the SGS intake group was significantly higher compared with the control group (*p* = 0.001) and that of *NAD(P)H quinone oxidoreductase 1* (*Nqo1*) and *glutathione peroxidase 3* (*Gpx3*) in the SGS intake group tended to be higher compared with the control group (*p* = 0.065 and 0.059, respectively. Figure 5A–D). In contrast, the above-mentioned NRF2 target genes were not altered in SAMP8 mice, but the expression of *glutamate-cysteine ligase modifier subunit* (*Gclm*) and *thioredoxin 1* (*Txn1*), which were other NRF2 target genes, were significantly higher in the SGS-fed group compared with the control group (*p* = 0.009 and 0.0446, respectively; Figure 5E,F). There were no significant differences in the expression of other anti-oxidative factors, such as *Catalase* (*Cat*), *Glutathione S-transferase Pi 1* (*Gstp1*), *Glutathione peroxidase 1* (*Gpx1*), *Gpx2, Superoxide dismutase 1* (*Sod1*), *Sod2*, *Glutamate-cysteine ligase catalytic subunit* (*Gclc*), *Thioredoxin reductase 1* (*Txnrd1*) and *Sulfiredoxin 1* (*Srxn1*) (Figure S3).

### 2.4. SGS Intake Transcriptionally Increases Mitochondrial Master Regulators in the Hippocampi of SAMP8 Mice

**Figure 6:**
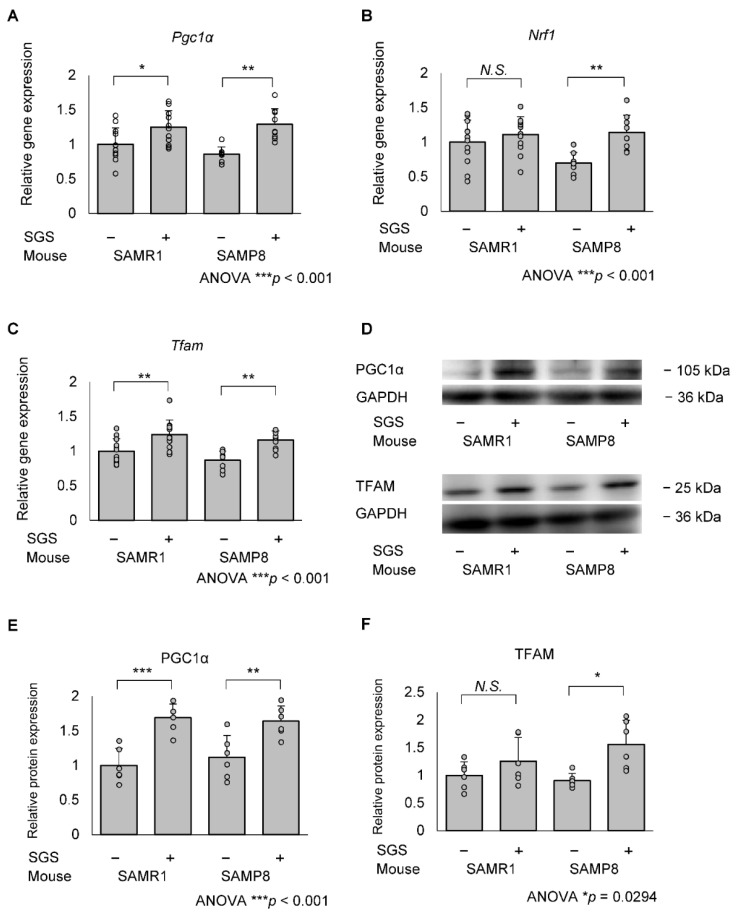
mRNA and protein expression of mitochondrial biogenesis-related genes in the SAMR1 or SAMP8 hippocampi. Mice were dissected at 13 months of age and mRNA and protein in the hippocampus were measured by qPCR and immunoblotting, respectively. The expression of mRNA in the control-treated SAMR1 mouse was set to 1 and the relative expression of the (**A**) *Pgc1*α, (**B**) *Nrf1* (**C**) *Tfam* genes was estimated using *Gapdh* as an internal control. The immunoblot analysis of PGC1α and TFAM are shown in (**D**). The relative protein expression of (**E**) Pgc1α and (**F**) Tfam were estimated (**A**–C) using Gapdh protein as an internal control. Mean values of SAMR1 mice fed with NC were set as 1 and relative protein expressions were shown as the means + SD of SAMR1/SGS− (*n* = 12), SAMR1/SGS+ (*n* = 12), SAMP8/SGS− (*n* = 8), SAMP8/SGS+ (*n* = 9). For protein expression, *n* = 6/group were analyzed. The data were analyzed by ANOVA with Tukey-Kramer’s post hoc test for comparison between differences. * *p* < 0.05, ** *p* < 0.01, *** *p* < 0.001, *N.S.*, not significant.

**Figure 7:**
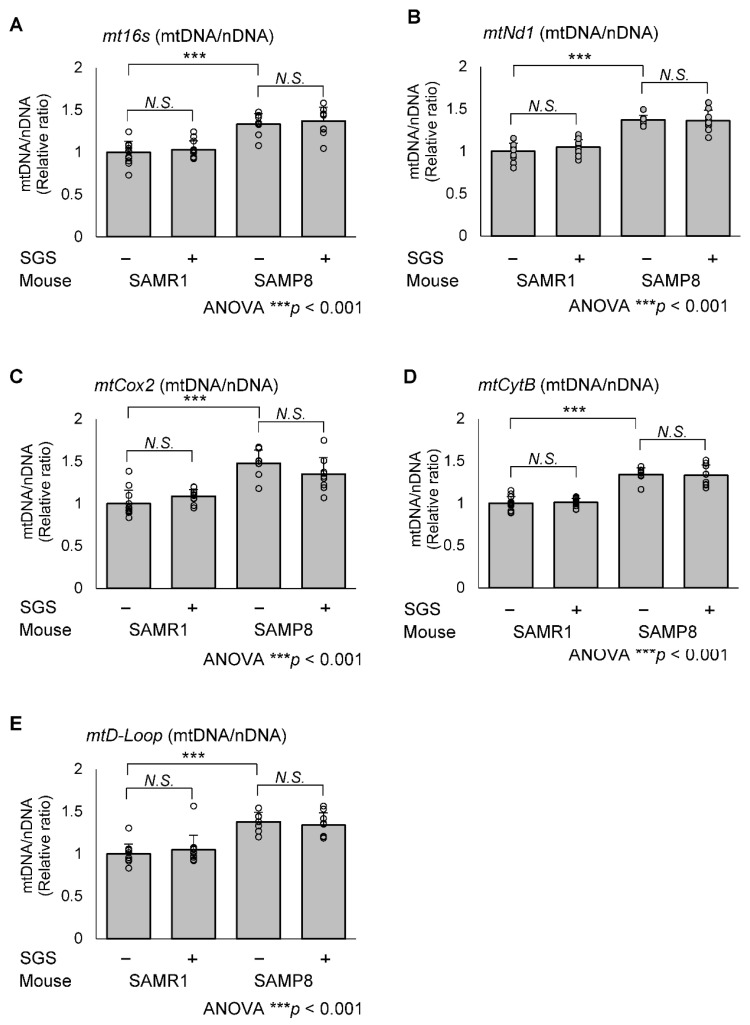
Relative mitochondrial copy number in the SAMR1 or SAMP8 hippocampi. The SAMR1 and SAMP8 mice were dissected at 13 months of age and hippocampal DNA was subjected to qPCR. The value of the control-treated SAMRA1 mouse was set to 1 and the relative mitochondrial DNA copy number (mtDNA-CN) was quantified by normalizing to nuclear DNA (nDNA). Primers for the mtDNA markers, (**A**) *mitochondrial 16s ribosomal RNA* (*mt16s*), (**B**) *NADH dehydrogenase 1* (*Nd1*), (**C**) *Cytochrome c oxidase 2* (*Cox2*)*,* (**D**) *Cytochrome b* (*mtCytB*) and (**E**) *Displacement loop* (*mtD-Loop*) the expression of and for nDNA marker *Hexokinase 2* (*HK2*) were used. Individual values of the mise are shown as open circles with means + SD of SAMR1/SGS− (*n* = 12), SAMR1/SGS+ (*n* = 12), SAMP8/SGS− (*n* = 8), and SAMP8/SGS+ (*n* = 9). The data were analyzed by ANOVA with Tukey-Kramer’s post hoc test for comparison between differences. *** *p* < 0.001, *N.S.*, not significant.

To evaluate whether SGS intake affects mitochondrial biogenesis, we measured the expression of *Pgc1α*, *Nrf1* and *Tfam* mRNA in the hippocampus. The mRNA expression levels of *Pgc1α* and *Tfam* in the SGS intake group were higher compared with the control group in both SAMR1 and SAMP8 mice (*p* = 0.036 for *Pgc1α* in SAMR1, *p* = 0.001 for *Pgc1α* in SAMP8, *p* = 0.0099 for *Tfam* in SAMR1, and *p* = 0.008 for *Tfam* in SAMP8; Figure 6A,C). *Nrf1* expression in the SGS intake group was significantly higher compared with the NC-fed group in the SAMP8 mice only (Figure 6B). Furthermore, PGC1α protein levels in the SGS-fed group were significantly higher compared with the control group in both SAMR1 and SAMP8 mice (*p* < 0.001 and 0.008, respectively; Figure 6E) and TFAM protein levels in the SGS-fed group were significantly higher compared with the control group in SAMP8 mice (*p* = 0.013; Figure 6F). To more directly evaluate whether SGS intake affects mitochondrial biogenesis, we measured mtDNA-CN levels. There was no significant difference in mtDNA-CN between the SGS-fed group and the control group for both SAMR1 and SAMP8 mice. Interestingly, in the NC-fed group, mtDNA-CN in SAMP8 mice was higher compared with the SAMR1 mice (Figure 7). TOMM40, which is a mitochondrial structural protein, was not altered in SAMR1 and SAMP8 mice, or between the SGS-fed and control groups (Figure S4), indicating no difference in mitochondrial mass. Furthermore, there were no significant differences in the expression of genes associated with mitochondrial fusion, *Mitofusin 1* (*Mfn1*), *Mitofusin 2* (*Mfn2*), and *Optic atrophy 1* (*Opa1*), or mitochondrial fission, *Dynamin-related protein 1* (*Drp1*) (Figure S5).

### 2.5. SGS Increases Mitochondria-Encoded Gene Expression in the Hippocampi of SAMP8 Mice

**Figure 8:**
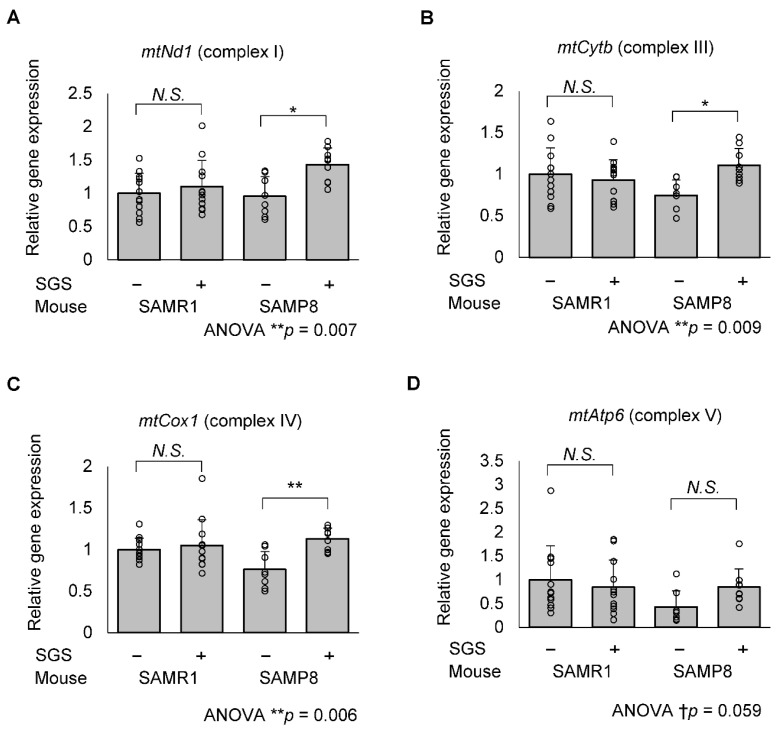
mRNA expression of respiratory chain complex genes encoded in the mitochondrial DNA of the SAMR1 and SAMP8 hippocampi. The SAMR1 and SAMP8 mice were dissected at 13 months of age and hippocampal mRNA was subjected to RT-qPCR. The mRNA expression of the control-treated SAMR1 mice was set to 1 and the relative mitochondrial mRNA expression of (**A**) *mtNd1* (complex I), (**B**) *mtCytb* (complex III), (**C**) *mtCox1* (complex IV) and (**D**) *ATPase6* (*mtAtp6*, complex V) was normalized to the housekeeping gene, *Gapdh*. Individual values of the mise are shown as open circles with means + SD of SAMR1/SGS− (*n* = 12), SAMR1/SGS+ (*n* = 12), SAMP8/SGS− (*n* = 8), and SAMP8/SGS+ (*n* = 9). The data were analyzed by ANOVA with Tukey-Kramer’s post hoc test for multiple comparisons. † *p* < 0.1, * *p* < 0.05, ** *p* < 0.01, *N.S.*, not significant.

We tested the possibility that increased PGC1αand TFAM promote the transcription of genes encoding mitochondrial DNA. The mRNA expression of *mitochondrial NADH dehydrogenase 1* (*mtNd1*, complex I), *mitochondrial cytochrome b* (*mtCytb*, complex III), *mitochondrial cytochrome c oxidase 1* (*mtCox1*, complex IV) in the SGS-fed group were significantly higher compared with those of the control group in SAMP8 mice; however, *mitochondrial ATPase6* (*mtAtp6*, complex V) mRNA expression levels tended to be increased (*p* = 0.022, 0.023, 0.006 and 0.414, respectively; Figure 8). In SAMR1 mice, there was no significant change in mRNA expression of these mitochondrial complex subunits (Figure 8).

## 3. Discussion

In the present study, we demonstrated for the first time that NRF2-activating SGS ameliorates age-related cognitive decline in SAMP8 mice. SGS intake conferred significantly improved long-term memory on SAMP8 mice (Figure 4B). Although it increased long-term memory in SAMR1 mice, the difference failed to reach statistical significance. SGS increased the expression of NRF2 target genes in both SAMR1 and SAMP8 mice; however, the responsive genes were different between strains. In contrast, SGS intake increased the levels of the mitochondrial master regulators, PGC1α and TFAM, in both strains, although *Nrf1* expression was increased only in SAMP8 mice. Accordingly, increased gene expression of the mitochondrial respiration complexes was observed in SAMP8, but not in SAMR1 mice. These results suggest that SGS intake may prevent age-related cognitive decline of senescence-accelerated mice (i.e., SAMP8 mice) through the enhancement of mitochondrial activity.

In the present study, the PA test showed that SGS intake conferred a significantly increased latency period on SAMP8 and tended to increase latency period on SAMR1 mice (Figure 4B). This result suggests that daily SGS intake may prevent age-related decline of long-term memory. In contrast, SGS intake showed no significant difference in the YM test in SAMR1 and SAMP8 mice (Figure 3B,C). Most likely, short-term memory did not decline at 8 or 10 months of age and no preventive effect of SGS appeared at these times. Consistent with this expectation, the NC-fed SAMP8 and SAMR1 group showed no significant difference (*p* = 0.495 at 8 months of age and *p* = 0.347 at 10 months of age). Previous reports demonstrated that 4- and 8-month-old SAMP8 mice exhibited obvious cognitive deficits compared with age-matched SAMR1 mice in a Morris-water maze test [26], whereas memory and spatial learning in the normal YM test was not significantly impaired until 12 months of age in SAMP8 mice [27]. A longer administration period would be necessary to clarify the protective effect of SGS on short-term memory. 

Mitochondrial biogenesis, one of the important functions of the mitochondria, is regulated by crosstalk with its major regulators, PGC1α and NRF2 [11,12]. PGC1α acts as an upstream regulator, a downstream target gene, and a coactivator of NRF2. It was previously reported that oral SFN intake increases the mRNA and protein levels of *Pgc1α*, *Nrf1,* and *Tfam* in the liver of high-fat induced non-alcoholic fatty liver disease (NAFLD) model rats and in a free fatty acid-stimulated human hepatocyte (HHL-5) cell line [28]. We demonstrated that SGS intake increased a similar pathway in both SAMR1 and SAMP8 mice; however, *Nrf1* expression was increased by SGS only in the SAMP8 hippocampus. Unexpectedly, mitochondrial biogenesis estimated by mtDNA-CN was increased in SAMP8 mice, but not affected by SGS treatment (Figure 7). TOMM40, which is a mitochondrial structural protein, was not altered in SAMR1 and SAMP8 mice, or between the SGS-fed and control groups (Figure S4), indicating no difference in mitochondrial mass. In a previous report, ROS production by mild respiration defects resulted in a compensatory increase of mtDNA-CN [29] and another report demonstrated that young SAMP8 mice exhibited reduced complex I activity compared with SAMR1 mice [30]. Furthermore, the respiratory function in SAMP8 mice was reported to decrease with age [31,32]. These findings and our results suggest that reduced mitochondrial function may lead to a compensatory increase of mtDNA-CN in the SAMP8 hippocampus. The SAMP8-specific predisposition to mitochondrial deterioration may contribute to the more pronounced effects of SGS on SAMP8 mice compared with SAMR1 mice. Of note, SGS intake-induced mitochondrial-encoded gene expression without an increase of mtDNA, may occur through the enhancement of mtDNA transcription.

Sulforaphane accumulates in the brain by crossing the blood-brain barrier, albeit slightly, compared with the liver, kidney, lung, and prostate [33]. In the present study, we demonstrated that the expression of the NRF2 target genes, *Hmox1*, *Nqo1*, and *Gpx3*, were increased in the SAMR1 hippocampus (Figure 5). However, it appeared that this gene expression profile was not significantly altered in SAMP8, leading to the alternative increase of *Gclm* and *thioredoxin* expression. It was previously reported that nuclear NRF2 levels were reduced in SAMP8 mice compared with the SAMR1 hippocampus [21,34] and this may be responsible for the difference between SAMP8 and SAMR1. *Pgc1α* and *Nrf1* are also known as NRF2 target genes and *Pgc1α* is induced in an NRF2-dependent manner [12,35]. *Nrf1* possesses a functional ARE in its promoter [36]. In the case of *Pgc1α*, it also contains a putative ARE, but whether it is functional or not has not been determined [11]. To prove a cause-effect relationship, further study is needed using knockdown or knockout analyses of these pathways. PGC1α also acts as an upstream regulator and induces *Nfe2l2* gene expression [11,37]; however, SGS-induced PGC1α did not result in *Nfe2l2* expression in our study (Figure 5A).

Previous reports indicated that PGC1α protein levels in the skeletal muscle of SAMP8 mice were decreased at 9 months compared with 5 months of age [26]; However, we did not observe significant differences in the mRNA or protein expression levels of *Pgc1α* between SAMP8 and SAMR1 mice. This indicates that the SAMP8 mice were in an early stage of senescence and may have retained a compensatory ability to maintain mitochondrial homeostasis. Mitochondrial dynamics are balanced by mitochondrial fusion and fission. The former fuses damaged mitochondria and compensates for mitochondrial functional defects [38]. In the present study, neither SGS intake nor mouse strain affected the transcriptional levels of genes involved in mitochondrial fusion and fission. These results suggest that mitochondrial dynamics were maintained in SAMP8 mice, although this should be verified by detecting mitochondrial dynamics or distribution in future studies.

There are several limitations that have not been mentioned above. First, hippocampus tissue includes several types of cells including neurons, astrocytes, oligodendrocytes, and microglia, whereas SGS target cells remain unknown. Future studies should focus on identifying how individual cell types are differentially affected by SGS. Second, the effect of SGS on long-term memory function before 12 months of age and on short-term memory function after 10 months of age remain unknown. More detailed and long-term observations of cognitive function are needed in the future to understand at which growth stages SGS affects cognitive function. Third, it has been reported that SGS and NRF2 activators have various functions, such as antioxidative, anti-neuroinflammation, detoxication activities, metabolism-improving functions [39], and it has been suggested that age-related cognitive decline may be the result of various factors [40]. SGS may prevent age-related cognitive decline by mechanisms other than those revealed in this study. Further multifaceted studies are needed to elucidate the full picture of the preventive effect of SGS on age-related cognitive decline.

In conclusion, the results of this study indicate that SGS upregulates PGC1α, NRF-1, and TFAM in the hippocampus and prevents age-related cognitive decline. Another clinical study demonstrated that supplementation with a dietary dose of SGS (30 mg/day) for 12 weeks significantly improved processing speed and working memory performance [20]. Consequently, SGS may be a suitable agent to prevent age-related cognitive decline, neurodegenerative disease, or other diseases that are caused, at least in part, by mitochondrial dysfunction.

## 4. Materials and Methods

### 4.1. Glucoraphanin Preparation

SGS was prepared as previously described [22]. BS extract powder was obtained from the KAGOME Co., Ltd. (Tokyo, Japan). BS was grown from seeds (Caudill Seed Co. Inc., Louisville, KY, USA) for 1 day following germination. Then, the 1-day-old BS was added to boiling water and incubated at 95 °C for 30 min and the sprout residues were removed by filtration. The boiling extract was mixed with dextrin and then spray-dried to yield BS extract powder containing 135 mg (approximately 310 μmol) of SGS per gram [41].

### 4.2. Animals and Glucoraphanin Preparation

Eight-weeks old male wild-type C57BL/6J mice were purchased from CLEA Japan, Inc (Tokyo, Japan) at 7 weeks of age and maintained in temperature- and humidity-controlled rooms with a 12-h light/dark cycle. Wild-type C57BL/6J mice were fed ad libitum with normal chow (NC, MF diet, Oriental Yeast, Tokyo, Japan) or NC supplemented with 0.3% SGS (containing 2.2% *w*/*w* BS extract powder), myrosinase (1.0% *w*/*w* mustard powder, #Y38, Minokyu Co., Ltd., Aichi, Japan), or both SGS and myrosinase for a week and expression levels of NRF2 target genes of the intestine or liver were quantified by qPCR.

One-month-old male SAMP8/TaSlc or SAMR1/TaSlc mice were purchased from Japan SLC (Hamamatsu, Japan) at 4 weeks of age and maintained in temperature- and humidity-controlled rooms with a 12-h light/dark cycle. Mice representing each strain were divided into two groups (*n* = 12), so that there was no difference in average body weight between the groups, and were acclimated for one week. Each group were fed ad libitum with normal chow (NC, MF diet, Oriental Yeast, Tokyo, Japan) or NC supplemented with 0.3% SGS (containing 2.2% *w*/*w* BS extract powder) and myrosinase (1.0% *w*/*w* mustard powder, #Y38, Minokyu Co., Ltd., Aichi, Japan) for 49 weeks. Myrosinase was added because it increases the bioavailability of sulforaphane [42]. All food were provided in powder form. We used powdered MF diet, and when BS extract powder and mustard powder were added to MF diet, they were stirred and mixed thoroughly. Mice were housed in 3 mice/cage with 2 food jars filled with NC or SGS-supplemented diet and allowed free access to food. The food jars were occasionally refilled with food to avoid emptying. The mice were subject to a modified Y-maze (YM) test at 8 and 10 months of age and a step-through passive avoidance (PA) test at 12 months of age. They were subsequently dissected at 13 months of age. The experimental procedures using animals was approved by the laboratory animal committee of Hirosaki University Graduate School of Medicine (permission number: M18008 and M18009).

### 4.3. Modified YM Test

To evaluate short-term memory function, the modified YM test was selected because of its low stress and invasiveness. The modified YM test was performed as described previously [43,44]. Testing occurs in a Y-shaped maze with three white, opaque plastic arms at a 120° angle from one another. This test consisted of two trial tasks, which included a sample phase trial and a subsequent test phase trial. The sample phase was performed with one of the three arms closed, allowing the mouse to explore freely within the arms for 5 min. Thirty minutes after the sample phase trial, the test phase trial was conducted with all arms opened, again allowing again the mouse to explore freely within the arms for 5 min. The arm that was closed in the sample phase was opened in the test phase and defined as a novel arm (Figure 3A). The mouse behavior was recorded to analyze the duration in the novel arm, which is indicative of short-term memory.

### 4.4. Step-Through PA Test

To evaluate long-term memory function, we used the PA test, which is widely used and well proven. The PA test was performed as a modified version of the previously reported method [45,46]. Briefly, the test was performed using a shuttle box, a device that consists of a bright box and a dark box. The bright and dark boxes were separated by a guillotine shutter and the floor of the dark box was able to conduct an electrical current. This test consisted of two trial tasks, which included a training phase and a subsequent test phase trial. In the training phase, the mice were placed in the bright box with the guillotine shutter closed and were allowed to explore the bright box. After 60 s, the guillotine shutter was released and as soon as the mice entered the dark box, the guillotine shutter was lowered and an electric shock was applied for 2 s. The mouse was kept in the dark box for 30 s and then returned to the home cage. The test phase was initiated 24 h after the training phase. The mice were placed into the bright box again in the test phase, and the elapsed time before entering the dark box was considered the latency time to evaluate long-term memory (Figure 4A). 

### 4.5. Quantitative RT-PCR

The brains were harvested and immediately frozen in liquid nitrogen. Total RNA along with protein and DNA were prepared using the AllPrep DNA/RNA/Protein mini kit (Qiagen, Venlo, The Netherlands) following the manufacturer’s protocol. Complementary DNA was synthesized using the PrimeScript™ RT reagent kit (TaKaRa Bio Inc., Shiga, Japan) using total RNA and iCycler (BIO-RAD, Hercules, CA, USA). Quantitative PCR was performed using the primer sets listed in Table 1. TB Green^®^ Premix Ex Taq™ II (TaKaRa Bio Inc.) and Applied Biosystems TM 7500 Fast (Thermo Fisher Scientific, Waltham, MA, USA) reagents were used. The qPCR reaction proceeded as follows: initial denaturation for 30 s at 95 °C, then 45 cycles of denaturation (5 s, 95 °C), annealing and extension (34 s, 60 °C). The relative mRNA expression was estimated by the ΔΔCt method using the housekeeping gene, *Glyceraldehyde-3-phosphate dehydrogenase* (*Gapdh*) as a reference. 

### 4.6. Immunoblot Analysis

Protein samples were prepared using the AllPrep DNA/RNA/Protein mini kit (Qiagen) following the manufacturer’s protocol. The protein samples were boiled in Laemmli’s sample buffer containing complete, EDTA-free protease inhibitor cocktail (Roche, Mannheim, Germany) with 5% 2-mercaptoethanol. The boiled protein samples were separated on SDS-PAGE gels and transferred to a polyvinylidene difluoride (PVDF) membranes (Millipore, Billerica, MA, USA). The membranes were blocked with 5% skim milk for PGC1α and GAPDH antibodies, 3% skim milk for TOMM40 antibody, or 2% skim milk for the TFAM antibody. Then, the blocked membrane was incubated with PGC1α antibody (1:1000; ab54481, Abcam, Cambridge, UK), TFAM antibody (1:3000, 22586-1-AP, Proteintech, Rosemont, IL, USA), TOMM40 antibody (1:1000; ab272921, Abcam), or GAPDH antibody (1:20,000; 10494-1-AP, Proteintech) overnight at 4 °C, followed by a 1-h incubation with HRP-conjugated goat anti-rabbit IgG (H+L) secondary antibody (1:10,000; 5220-0458, SeraCare Life sciences Inc., Milford, MA, USA) at room temperature. Immobilon Forte Western HRP Substrate (Millipore) was used for chemiluminescent detection. The immunoreactive band intensities were measured using Image J software and normalized to GAPDH.

### 4.7. Mitochondrial DNA Copy Number Analysis 

The mitochondrial DNA copy number (mtDNA-CN) analysis was performed as described previously [47]. The mtDNA and nuclear DNA (nDNA) were prepared using the AllPrep DNA/RNA/Protein mini kit (Qiagen), following the manufacturer’s protocol. The extracted DNA was subjected to qPCR in the same manner as cDNA using the primer sets listed in Table 2. The relative mtDNA-CN was quantified using primers for the mitochondrial genes, *16s ribosomal RNA* (*mt16s*), *NADH dehydrogenase 1* (*Nd1*), *Cytochrome c oxidase 2* (*Cox2*), *Cytochrome b* (*mtCytB*), and *Displacement loop* (*mtD-Loop*), and were normalized by nDNA abundance using primers for *Hexokinase 2* (*HK2*).

### 4.8. Statistical Analysis

A paired *t*-test was used for before after comparisons. A one-way ANOVA with Tukey-Kramer’s post hoc test was used to analyze differences in multiple comparisons. The data are presented as means with standard deviation (SD) values. Statistical significance was considered at *p* < 0.05. For the behavioral analysis (the YM test and the PA test), because individual differences were very large, an exclusion test was performed for each group, and a statistical analysis was performed by excluding the applicable values. We used R (ver. 3.6.3) with R Studio (ver. 1.2.5033) to evaluate the data.

## Figures and Tables

**Table 1:** Primer sets used to measure the mRNA of the indicated genes by qPCR.

Gene	Forward (5′-3′)	Reverse (5′-3′)
*Nfe2l2*	CAGCACATCCAGACAGACACCA	CGTATTAAGACACTGTAACTCGGGAATGG
*Hmox1*	TGACACCTGAGGTCAAGCAC	TCCTCTGTCAGCATCACCTG
*Nqo1*	AGCGTTCGGTATTACGATCC	AGTACAATCAGGGCTCTTCTCG
*Gpx3*	CGAGTATGGAGCCCTCACCA	GCCCAGAATGACCAAGCCAA
*Gclm*	GGGAACCTGCTCAACTGGGG	CTGCATGGGCATGGTGCATT
*Txn1*	CCCTTCTTCCATTCCCTCT	TCCACATCCACTTCAAGGAAC
*Pgc1α*	CACCGCAATTCTCCCTTGTA	TGCGGTATTCATCCCTCTTG
*Nrf1*	CCTCTGATGCTTGCGTCGTCT	TTACTCTGCTGTGGCTGATGG
*Tfam*	TGGAGGGAGCTACAGAAGCAG	GCCTCCTTCTCCATACCCATCAGC
*Cat*	GGCAAAGGTGTTTGAGCATATT	GAGTCTGTGGGTTTCTCTTCTG
*Gsta1/3*	GGCAGAATGGAGTGCATCA	TCCAAATCTTCCGGACTCTG
*Gstm3*	GCACAACCTGTGTGGAGAGAC	ACTCTGGCTTCTGCTTCTCAA
*Gstp1*	TGTCACCCTCATCTACACCAAC	GGACAGCAGGGTCTCAAAAG
*Gpx1*	ATGCCTTAGGGGTTGCTAGG	CGACATCGAACCCGATATAGA
*Gpx2*	CAGCTTCCAGACCATCAACA	CACTGAGCCCTGAGGAAGAC
*Sod1*	AACCAGTTGTGTTGTCAGGAC	CCACCATGTTTCTTAGAGTGAGG
*Sod2*	CAGACCTGCCTTACGACTATGG	CTCGGTGGCGTTGAGATTGTT
*Gclc*	TGGCCACTATCTGCCCAATT	GTCTGACACGTAGCCTCGGTAA
*Txnrd1*	GTGGCGACTTGGCTAATC	ACCAGGAGAGACACTCAC
*Srxn1*	CCCACTGGACCAACTTCTGT	GTGGCTAGCTCAGACCAAGG
*Mfn1*	GCAGACAGCACATGGAGAGA	GATCCGATTCCGAGCTTCCG
*Mfn2*	TGCACCGCCATATAGAGGAAG	TCTGCAGTGAACTGGCAATG
*Opa1*	ACCTTGCCAGTTTAGCTCCC	TTGGGACCTGCAGTGAAGAA
*Drp1*	ATGCCAGCAAGTCCACAGAA	TGTTCTCGGGCAGACAGTTT
*mtNd1*	TACGAGCCGTAGCCCAAACA	GATCGTAACGGAAGCGTGGA
*mtCytb*	ATTCCTTCATGTCGGACGAG	ACTGAGAAGCCCCCTCAAAT
*mtCox1*	CTGAGCGGGAATAGTGGGTA	TGGGGCTCCGATTATTAGTG
*mtAtp6*	TCCCATCCTCAAAACGCCTA	CCAGCTCATAGTGGAATGGC
*Gapdh*	AGGTCGGTGTGAACGGATTTG	TGTAGACCATGTAGTTGAGGTCA

Abbreviations: Nfe2l2, Nuclear Factor E2-Related factor 2; Hmox1, Heme oxygenase 1; Nqo1, NAD(P)H quinone dehydrogenase 1; Gpx1/2/3, Glutathione peroxidase 1/2/3; Gclm, Glutamate-cysteine ligase modifier subunit; Txn1, Thioredoxin 1; Pgc1αPeroxisome proliferator-activated receptor gamma coactivator-1 alpha;Nrf1, Nuclear respiratory factor-1; Tfam, Mitochondrial transcription factor A; Cat, Catalase; Gsta1/3, Glutathione S-Transferase alpha 1/3; Gstm3, Glutathione S-transferase Mu 3; Gstp1, Glutathione S-transferase Pi 1; Sod1/2, Superoxide dismutase 1/2; Gclc, Glutamate-cysteine ligase catalytic subunit;Txnrd1, Thioredoxin reductase 1; Srxn1, Sulfiredoxin 1; Mfn1, Mitofusin 1; Mfn2, Mitofusin 2; Opa1, Optic atrophy 1; Drp1, Dynamin-related protein 1; mtNd1, Mitochondrial NADH dehydrogenase 1; mtCytb, Mitochondrial cytochrome b; mtCox1, mitochondrial cytochrome c oxidase 1; mtATP6, Mitochondrial ATPase6; Gapdh, Glyceraldehyde-3-phosphate dehydrogenase.

**Table 2:** Primer sets used to measure DNA of the indicated genes using qPCR.

Gene	Forward (5′-3′)	Reverse (5′-3′)
*mt16s*	CCGCAAGGGAAAGATGAAAGAC	TCGTTTGGTTTCGGGGTTTC
*mtNd1*	CTAGCAGAAACAAACCGGGC	CCGGCTGCGTATTCTACGTT
*mtCox2*	GTTGATAACCGAGTCGTT	CCTGGGATGGCATCAGTT
*mtCytb*	AGACAAAGCCACCTTGACCCGAT	ACGATTGCTAGGGCCGCGAT
*mtD-Loop*	TGCCCCTCTTCTCGCTCCGG	GGCGATAACGCATTTGATGGCC
*HK2*	GCCAGCCTCTCCTGATTTTAGTGT	GGGAACACAAAAGACCTCTTCTGG

Abbreviation: mt16s, mitochondrial 16s ribosomal RNA; mtNd1, mitochondrial NADH dehydrogenase 1; mtCox2, mitochondrial cytochrome c oxidase 2; mtCytb, mitochondrial cytochrome b; mtD-Loop, mitochondrial Displacement loop; Hk2, Hexokinase 2.

## Data Availability

The data that support the finding of this study are available from the corresponding author upon reasonable request.

## Supplementary Materials

The following are available online at: https://www.mdpi.com/article/10.3390/ijms23158433/s1.
