# Supplementary material for: Sulforaphane Increase Mitochondrial Biogenesis-Related Gene Expression in the Hippocampus and Suppresses Age-Related Cognitive Decline in Mice
Source: Int J Mol Sci. 2022 Jul 29;23(15):8433. doi: 10.3390/ijms23158433 (PMC9369397; doi:10.3390/ijms23158433)
Supplement: Supplementary file 1 [file ijms-23-08433-s001.zip › ijms-1814593-supplementary.pdf]

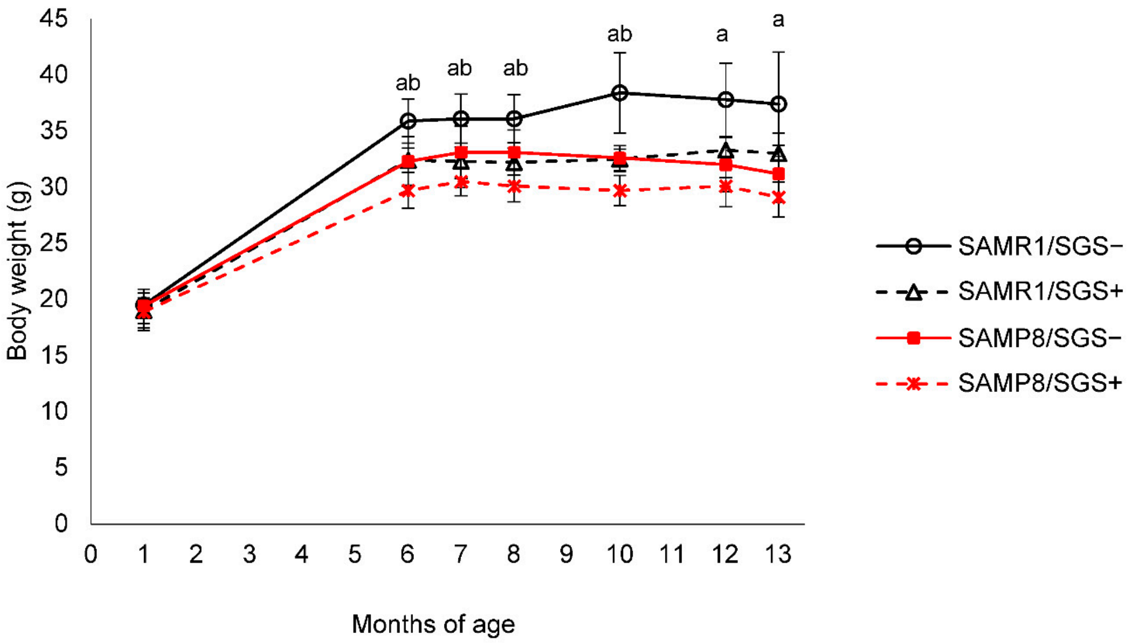


**Figure S1. The body weight changes of SAMR1 and SAMP8 mice during the experiment.**

SAMR1 and SAMP8 mice were fed with NC (SGS−) or SGS-containing diet (SGS+) (0.3% w/w) from 1 to 13 months of age. The data are shown as the means ± SD of SAMR1/SGS− (*n* = 12), SAMR1/SGS+ (*n* = 12), SAMP8/SGS− (*n* = 10), SAMP8/SGS+ (*n* = 9). The data at each time-point were analyzed by ANOVA with Tukey-Kramer post hoc test for multiple comparison. a: $p < 0.05$ for SGS− vs SGS+ in SAMR1, b: $p < 0.05$ for SGS− vs SGS+ in SAMP8.

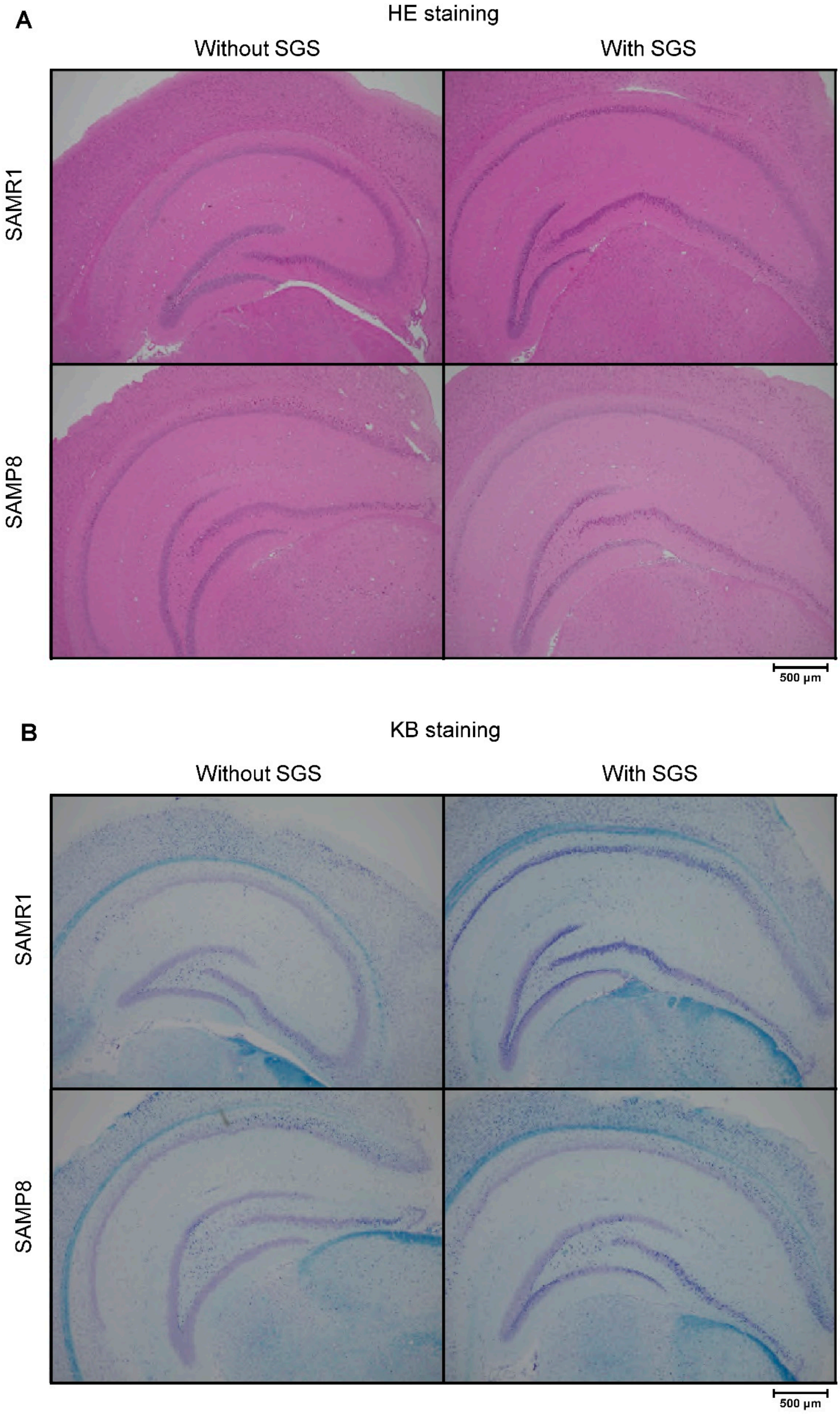


**Figure S2. Histological analysis of the hippocampal tissue section analysis in SAMR1 and SAMP8 mice.**

SAMR1 and SAMP8 mice were fed with or without SGS (0.3% w/w) from 1 to 13 months of age were dissected at 13 months of age. The hippocampus was fixed with formalin, paraffin-embedded, sliced and stained by (A) hematoxylin and eosin (HE) method or (B) Kluver-Barrera (KB) method.

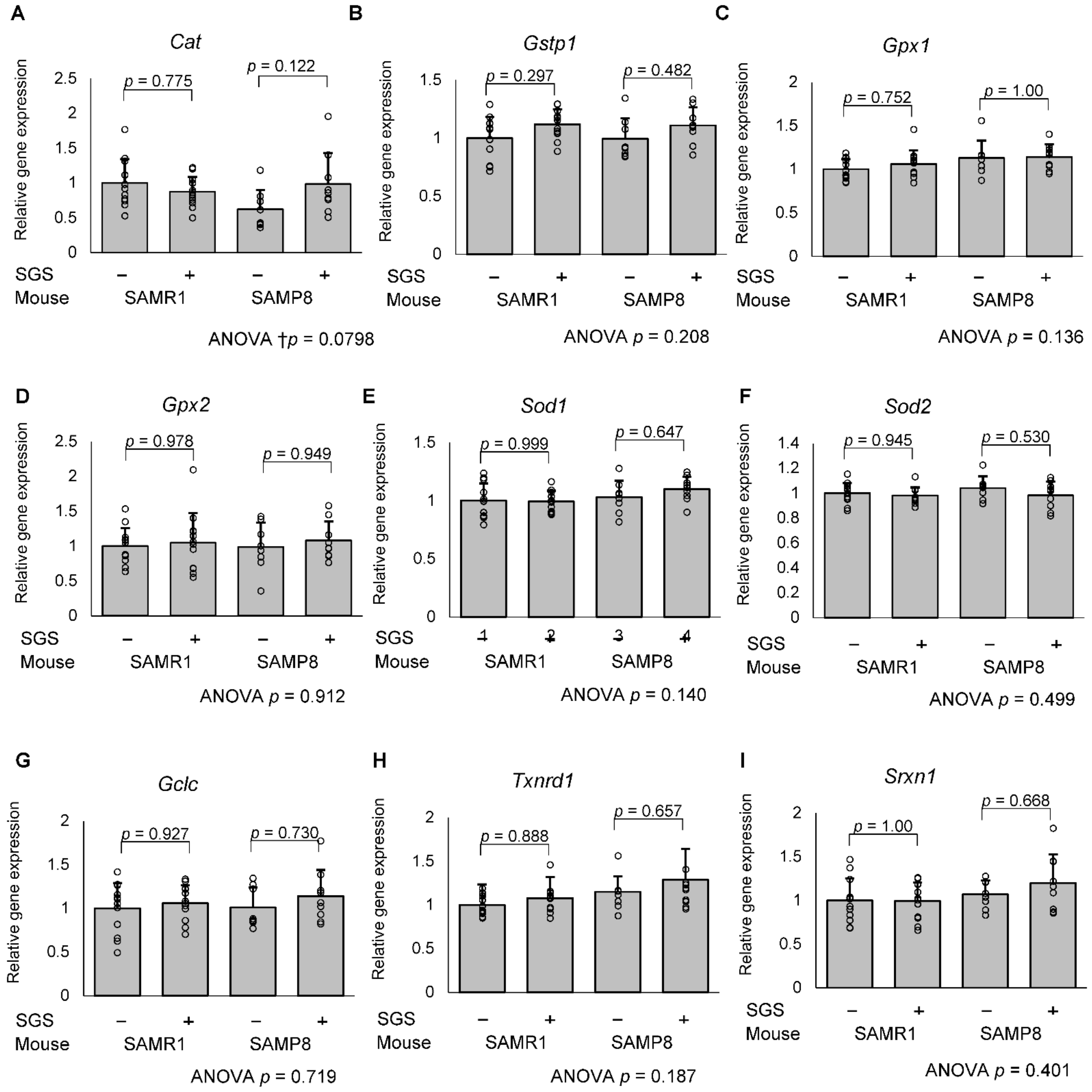


**Figure S3. The relative expression of NRF2/ARE pathway-regulated genes in SAMR1 and SAMP8 mice hippocampi.** SAMR1 and SAMP8 mice were fed with or without SGS (0.3% w/w) from 1 to 13 months of age and mice were dissected at 13 months of age. Total RNA extracted from the hippocampus were subjected to RT-qPCR. The value for control-fed SAMR1 mice was set to 1 and relative mRNA expression of (A) *Catalase (Cat)*, (B) *Glutathione S-transferase Pi 1 (Gstp1)*, (C) *Glutathione peroxidase 1 (Gpx1)*, (D) *Gpx2* (E) *Superoxide dismutase 1 (Sod1)*, (F) *Sod2*, (G) *Glutamate-cysteine ligase catalytic subunit (Gclc)*, (H) *Thioredoxin reductase 1 (Txnrd1)*, and (I) *Sulfiredoxin 1 (Srxn1)* were normalized by using *Glyceraldehyde-3-phosphate dehydrogenase (Gapdh)* as an internal control. Individual values of the mise are shown as open circles with means + SD of SAMR1/SGS− ($n$ = 12), SAMR1/SGS+ ($n$ = 12), SAMP8/SGS− ($n$ = 8), SAMP8/SGS+ ($n$ = 9). The data were analyzed by ANOVA with Tukey-Kramer post hoc test for multiple comparison.

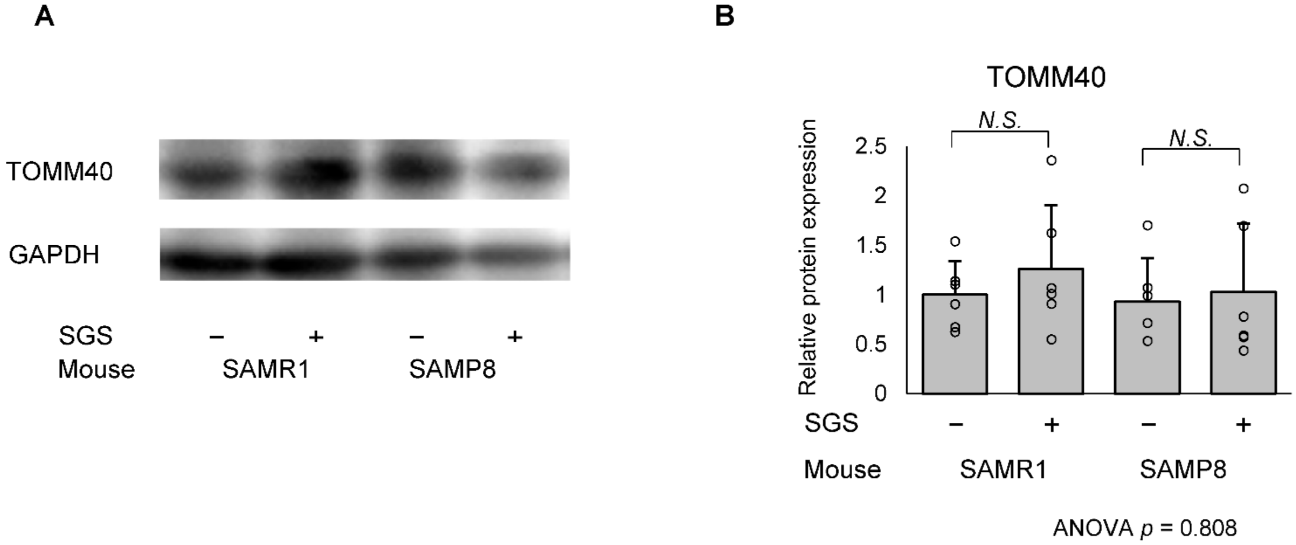


**Figure S4. Assessment of mitochondrial mass by TOMM40 expression in the SAMR1 or SAMP8 hippocampi.**

The SAMR1 and SAMP8 mice were dissected at 13 months of age. Proteins extracted from the hippocampus were measured by immunoblotting. The immunoblot of TOMM40 and GAPDH were shown in A. The value for control-fed SAMR1 mice was set to 1 and relative protein expressions of (B) TOMM40 were normalized by using GAPDH protein as an internal control. Individual values of the mise are shown as open circles with means + SD. For protein expression, $n$ = 6/group were analyzed. The data were analyzed by ANOVA with Tukey-Krammer post hoc test for comparison between different. *N.S.*, not significant.

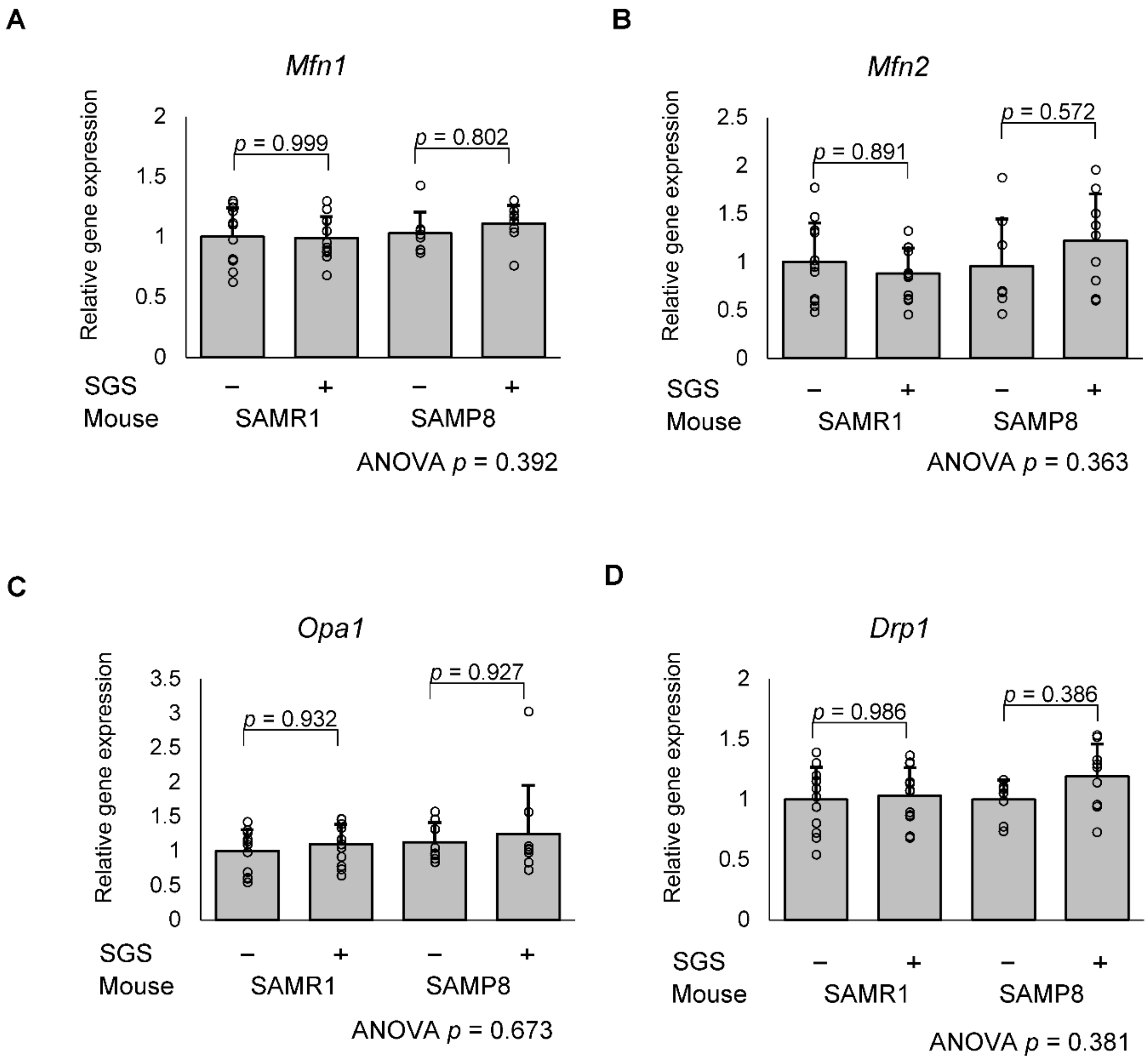


**Figure S5. The relative expression of mitofission and mitofusion related genes in SAMR1 and SAMP8 mice hippocampi.** SAMR1 and SAMP8 mice were fed with or without SGS (0.3% w/w) from 1 to 13 months of age and mice were dissected at 13 months of age. Total RNA extracted from the hippocampus were subjected to RT-qPCR. The value for control-fed SAMR1 mice was set to 1 and relative mRNA expression of (A) *Mitofusin 1 (Mfn1)* (B) *Mitofusin 2 (Mfn2)* (C) *Optic atrophy 1 (Opa1)* and (D) *Dynamin-related protein 1 (Drp1)* were estimated by Glyceraldehyde-3-phosphate dehydrogenase (Gapdh) as an internal control. Individual values of the mise are shown as open circles with means + SD of SAMR1/SGS- (n = 12), SAMR1/SGS+ (n = 12), SAMP8/SGS- (n = 8), SAMP8/SGS+ (n = 9). The data were analyzed by ANOVA with Tukey-Kramer post hoc test for multiple comparison.
